# A dataset of high-speed video recordings of wheel-rail traction enhancement using a full-scale testing platform

**DOI:** 10.1016/j.dib.2025.111806

**Published:** 2025-06-18

**Authors:** Sadaf Maramizonouz, Sadegh Nadimi, William A. Skipper, Stephen R. Lewis, Roger Lewis

**Affiliations:** a School of Engineering, Newcastle University, NE1 7RU, UK; b Leonardo Centre for Tribology, Department of Mechanical Engineering, University of Sheffield, Sheffield, S1 3JD, UK; c Rail Technologies Department, British Steel, Scunthorpe Rail and Section Mill, Brigg Road, Scunthorpe, DN16 1BP, UK

**Keywords:** Rail-sanding, Traction, Adhesion, Full-scale testing

## Abstract

In a process called rail-sanding, the acceleration and deceleration of train can be regulated by applying sand at the wheel-rail interface via an on-board system. A dataset of 78 high-speed video recordings of rail-sanding process using a full-scale testing platform is provided in this data note. The videos are recorded for various case studies, namely different positioning of the sander hose aiming at the rail, nip, and wheel with various angles, and different materials used as rail-sand. The velocities of sand particles is extracted from these high-speed videos using particle image velocimetry software. The spread angle of the particles as they flow out of the sander hose is also measured with the use of image processing software. The data extracted from these high-speed recording can be utilised for calibration, validation, and verification of experimental and numerical set-ups, as well as for training artificial intelligence models.

Specifications Table

**Table:** 

Subject	Railway engineering
Specific subject area	In railway industry, rail-sanding is used to mitigate wheel-rail traction loss by depositing sand particles into the wheel-rail interface in a stream of compressed air.
Type of data	High-Speed Video Recordings (.CINE and .AVI) Raw
Data collection	The full-scale testing platform used to carry out the experiments was originally developed by British Rail Research and later modified by and is currently located at the Leonardo Centre for Tribology – The University of Sheffield. The high-speed videos of the rail-sanding tests were recorded using high-speed cameras: (1) A Phantom V210 camera with the Phantom Camera Control software for the case studies of hose’s position and aim recorded at 750 fps, and (2) Two Daheng Imaging MER-050–560U3M cameras with 5MP 6 MM C-mount lenses and the Galaxy viewer software recording videos from both front and side views for the case studies of different granular materials recorded at 560 fps. The experiments were repeated at least three times and videos were recorded for each test. If during any experiment, the testing platform showed malfunction or unreasonable behaviours or if the value of entrainment efficiency was shown to deviate over ∼3 % compared to the average, the test was removed from the dataset.
Data source location	Leonardo Centre for Tribology, Department of Mechanical Engineering, University of Sheffield, Sheffield, S1 3JD, UK
Data accessibility	Repository name: Zenodo Data identification number: 10.5281/zenodo.10932119 10.5281/zenodo.10932746 10.5281/zenodo.10927364 Direct URL to data: https://doi.org/10.5281/zenodo.10932119 https://doi.org/10.5281/zenodo.10932746 https://doi.org/10.5281/zenodo.10927364
Related research article	S. Lewis, S. Riley, D. Fletcher, and R. Lewis, “Optimisation of a Railway Sanding System for Optimal Grain Entrainment into the Wheel–Rail Contact," Proceedings of the Institution of Mechanical Engineers, Part F: Journal of Rail and Rapid Transit, vol. 232, no 1, pp. 43–62, 2018, doi:10.1177/0954409716656220

## 1 Value of the Data

• A dataset of high-speed video recordings of rail-sanding processes
• Post-processing can provide detailed data such as particle velocities, trajectories, and spread angles
• Can be used to calibrate experimental platforms
• Can be used to validate numerical simulations
• Can be used to train artificial intelligence models

## 2 Background

One key area of research in railway industry is the study of the traction or adhesion^1^ between a train wheel and the rail. The aim of this research field is to improve railway transportation by controlling the wheel-rail traction especially during acceleration and decelerations phases of the train kinematics [[1], [2], [3]]. Due to several environmental factors such as the existence of third-body layers including moisture, oil, leaf, or any other contaminant on the top of the rail, wheel-rail traction levels may decline which can result in train delays, safety issues, and in some unfortunate cases train collisions [[4], [5], [6]].

Rail-sanding is used as a solution to mitigate traction loss by depositing sand particles into the wheel-rail interface in a stream of compressed air using a train-borne system [[5], [6], [7]]. This dataset presents 78 high-speed videos of rail-sanding processes recorded during various full-scale experiments, including Lewis et al. [1] study and more recently conducted tests. Post-processing of the high-speed video recordings provide more detailed data such as particle velocities, trajectories, and spread angles.

## 3 Data Description

All the video recordings are publicly available. The details of the high-speed video recordings of full-scale experiments of rail-sanding are shown in Table 1. Due to the size restrictions of the cloud-based data repositories, only selections of the videos (file names are distinguished in bold font in Table 1) are already uploaded. The rest of the video recordings are available upon request.

**Table 1:** High-speed video recordings of rail-sanding full-scale experiments for all the case studies of the sanding system (geometry and positioning is based on the work of Lewis et al. [1]).

Hose aimed at	Hose angle to rail *(°)*	Granular material used as rail-sand	File name	File type	View
Rail	15	GB rail-sand	**1–1–1**	.CINE	Side
		GB rail-sand	1–1–2	.CINE	Side
		GB rail-sand	1–1–3	.CINE	Side
	20	GB rail-sand	**1–1–4**	.CINE	Side
		GB rail-sand	1–1–5	.CINE	Side
		GB rail-sand	1–1–6	.CINE	Side
	25	GB rail-sand	**1–1–7**	.CINE	Side
		GB rail-sand	1–1–8	.CINE	Side
		GB rail-sand	1–1–9	.CINE	Side
	30	GB rail-sand	**1–1–10**	.CINE	Side
		GB rail-sand	1–1–11	.CINE	Side
		GB rail-sand	1–1–12	.CINE	Side
Nip	10	GB rail-sand	**1–2–1**	.CINE	Side
		GB rail-sand	1–2–2	.CINE	Side
		GB rail-sand	1–2–3	.CINE	Side
	15	GB rail-sand	**1–2–4**	.CINE	Side
		GB rail-sand	1–2–5	.CINE	Side
		GB rail-sand	1–2–6	.CINE	Side
	20	GB rail-sand	**1–2–7**	.CINE	Side
		GB rail-sand	1–2–8	.CINE	Side
		GB rail-sand	1–2–9	.CINE	Side
Wheel	5	GB rail-sand	1–3–7	.CINE	Side
		GB rail-sand	1–3–8	.CINE	Side
		GB rail-sand	1–3–9	.CINE	Side
	10	GB rail-sand	**1–3–1**	.CINE	Side
		GB rail-sand	1–3–2	.CINE	Side
		GB rail-sand	1–3–3	.CINE	Side
	20	GB rail-sand	**1–3–4**	.CINE	Side
		GB rail-sand	1–3–5	.CINE	Side
		GB rail-sand	1–3–6	.CINE	Side
Nip	10	GB rail-sand	**GB1_2024–02–15_11_15_26_675**	.AVI	Side
			**GB1H_2024–02–15_11_15_27_880**	.AVI	Front
			GB2_2024–02–15_14_43_36_418	.AVI	Side
			GB2H_2024–02–15_14_43_37_497	.AVI	Front
			GB3_2024–02–15_15_29_26_672	.AVI	Side
			GB3H_2024–02–15_15_29_27_898	.AVI	Front
	10	RCG 2.0 mm	**RG2mm1_2024**–**02–16_11_44_27_680**	.AVI	Side
			**RG2mm1H_2024**–**02–16_11_44_28_899**	.AVI	Front
			RG2mm2_2024–02–16_13_31_48_196	.AVI	Side
			RG2mm2H_2024–02–16_13_31_49_016	.AVI	Front
			RG2mm3_2024–02–16_13_57_28_994	.AVI	Side
			RG2mm3H_2024–02–16_13_57_30_827	.AVI	Front
	10	RCG 1.18 mm	**RG1.18mm1_2024**–**02–16_15_06_26_109**	.AVI	Side
			**RG1.18mm1H_2024**–**02–16_15_06_27_281**	.AVI	Front
			RG1.18mm2_2024–02–16_15_28_24_482	.AVI	Side
			RG1.18mm2H_2024–02–16_15_28_26_147	.AVI	Front
			RG1.18mm3_2024–02–16_15_48_35_944	.AVI	Side
			RG1.18mm3H_2024–02–16_15_48_37_093	.AVI	Front
	10	RCG 0.6 mm	**RG0.6mm1_2024**–**02–19_11_33_09_657**	.AVI	Side
			**RG0.6mm1H_2024**–**02–19_11_33_10_525**	.AVI	Front
			RG0.6mm2_2024–02–19_11_53_29_723	.AVI	Side
			RG0.6mm2H_2024–02–19_11_53_30_989	.AVI	Front
			RG0.6mm3_2024–02–19_12_12_52_250	.AVI	Side
			RG0.6mm3H_2024–02–19_12_12_53_362	.AVI	Front
	10	Dolomite	**DOL1_2024**–**02–20_15_40_43_836**	.AVI	Side
			**DOL1H_2024**–**02–20_15_40_44_786**	.AVI	Front
			DOL2_2024–02–20_15_58_29_879	.AVI	Side
			DOL2H_2024–02–20_15_58_30_780	.AVI	Front
			DOL3_2024–02–20_16_12_44_547	.AVI	Side
			DOL3H_2024–02–20_16_12_45_660	.AVI	Front
	10	Noncoated Alumina	**DUC1_2024**–**02–20_14_21_53_665**	.AVI	Side
			**DUC1H_2024**–**02–20_14_21_54_906**	.AVI	Front
			DUC2_2024–02–20_14_42_02_056	.AVI	Side
			DUC2H_2024–02–20_14_42_03_251	.AVI	Front
			DUC3_2024–02–20_15_06_22_805	.AVI	Side
			DUC3H_2024–02–20_15_06_23_624	.AVI	Front
	10	Coated Alumina	**D1_2024**–**02–19_15_04_54_427**	.AVI	Side
			**D1H_2024**–**02–19_15_04_55_667**	.AVI	Front
			D2_2024–02–19_15_39_27_013	.AVI	Side
			D2H_2024–02–19_15_39_28_575	.AVI	Front
			D3_2024–02–19_16_04_53_430	.AVI	Side
			D3H_2024–02–19_16_04_54_724	.AVI	Front
			D4_2024–02–20_10_55_42_889	.AVI	Side
			D4H_2024–02–20_10_55_43_994	.AVI	Front
			D5_2024–02–20_11_21_58_335	.AVI	Side
			D5H_2024–02–20_11_21_59_410	.AVI	Front
			D6_2024–02–20_11_42_56_614	.AVI	Side
			D6H_2024–02–20_11_42_57_528	.AVI	Front

For the case studies of the hose position and aim, the high-speed videos are presented as .CINE files and for the case studies of seven different granular materials used as rail-sand, the high-speed videos are presented as .AVI files.

The video files of the sander hose’s position case studies namely files 1–1–1, 1–1–4, 1–1–7, and 1–1–10 for the hose aimed at rail are available on Zenodo repository with the doi:10.5281/zenodo.10932119 [8]. Files 1–2–1, 1–2–4, and 1–2–7 for the hose aimed at nip, and files 1–3–1 and 1–3–4 for the hose aimed at wheel are available on Zenodo repository with the doi:10.5281/zenodo.10932746 [9]. For the case studies of the seven granular material candidates, video files from side and front views including GB1 and GB1H for Great Britain rail-sand, RG2mm1 and RG2mm1H for 2 mm recycled cushed glass, RG1.18mm1 and RG1.18mm1H for 1.18 mm recycled crushed glass, RG0.6mm1 and RG0.6mm1H for 0.6 mm recycled crushed glass, D1 and D1H for coated alumina, DUC1 and DUC1H for non-coated alumina, and DOL1 and DOL1H for dolomite are available on Zenodo repository with the doi:10.5281/zenodo.10927364 [10].

## 4 Experimental Design, Materials and Methods

### 4.1 Full-scale testing platform

**Fig. 1:**
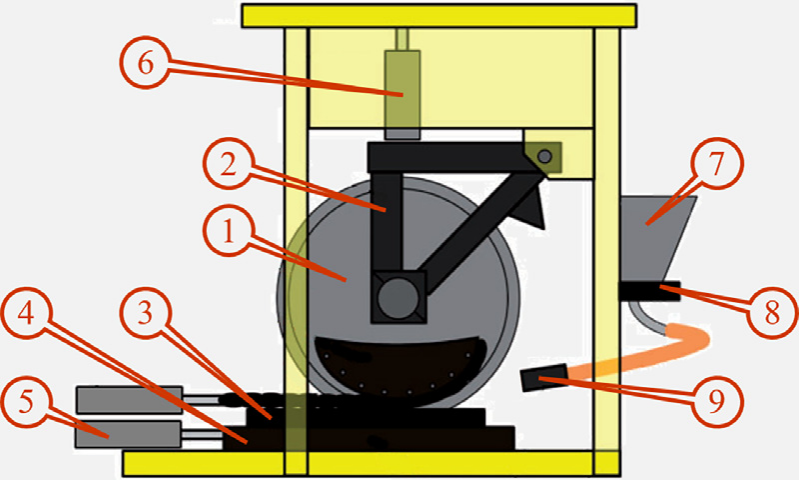
Schematic of the full-scale rail-sanding testing platform including **(1)** wheel, **(2)** load frame, **(3)** rail, **(4)** slider bed, **(5)** horizontal actuator, **(6)** vertical actuator, **(7)** sand hopper, **(8)** sand valve, and **(9)** sander hose [1].

The full-scale testing platform used to carry out the experiments was originally developed by British Rail Research and later modified for the present experiments by and is currently located at the Leonardo Centre for Tribology – The University of Sheffield [1]. A schematic of the testing platform is presented in Fig. 1.

The diameter of the wheel (1) and the length and width of the rail (3) are 1016 mm, 1010 mm, and 70 mm, respectively. The wheel is mounted on a rigid axle and is free to rotate due to the friction at the wheel/rail interface. The axle can also rotate within two journal bearings positioned in a load frame (3) pivoted at one end. This allows raising and lowering of the wheel to sit on the rail. The axle weight the wheel experiences is reproduced through a vertical actuator (6) which can elevate the wheel and place it at the start of the cycle as well. The rail is installed on a linear slider bed (4) and a horizontal actuator (5) is used to pull the rail and slider bed. The hydraulics and motion of the test rig are controlled via computer using National Instruments LabVIEW. The sand hopper (7) is filled with rail-sand and mounted to the frame of the test rig. A sand valve (8) is placed at the bottom of the sand hopper controls the flow of the sand to the sander hose. The sander hose has an internal bore diameter of 25 mm. Some of the tests reported in [1] also looked at the effect of using a nozzle with a 20 mm bore inserted into the end of the hose. The hose (9) has a length and outer diameter of 150 mm and 25 mm, respectively. For more information on the test rig setup and function please refer to the work by Lewis et al. [1].

### 4.2 Rail-sanding experiments

The experiments are performed by positioning the rail towards the far right-hand side of the test rig (Fig. 1). Then the wheel is lowered on the top of the rail and the rail starts to move towards the furthest left of the test rig (Fig. 1) with a set velocity while the sand is deposited on the wheel-rail interface with a flow rate of typically ∼0.03 kg/s. Due to the friction between the wheel and the rail, the wheel also rotates on the top of the rail in clockwise direction with a rotational velocity of ∼0.095 rad/s. At the end, the motion is stopped and the sand remaining on top of the rail is collected to measure the entrainment efficiency by calculating the ratio of the mass of the crushed sand collected from top of the rail to the total mass of the sand used for each experiment. For more detailed information on the experiments please refer to [1].

**Fig. 2:**
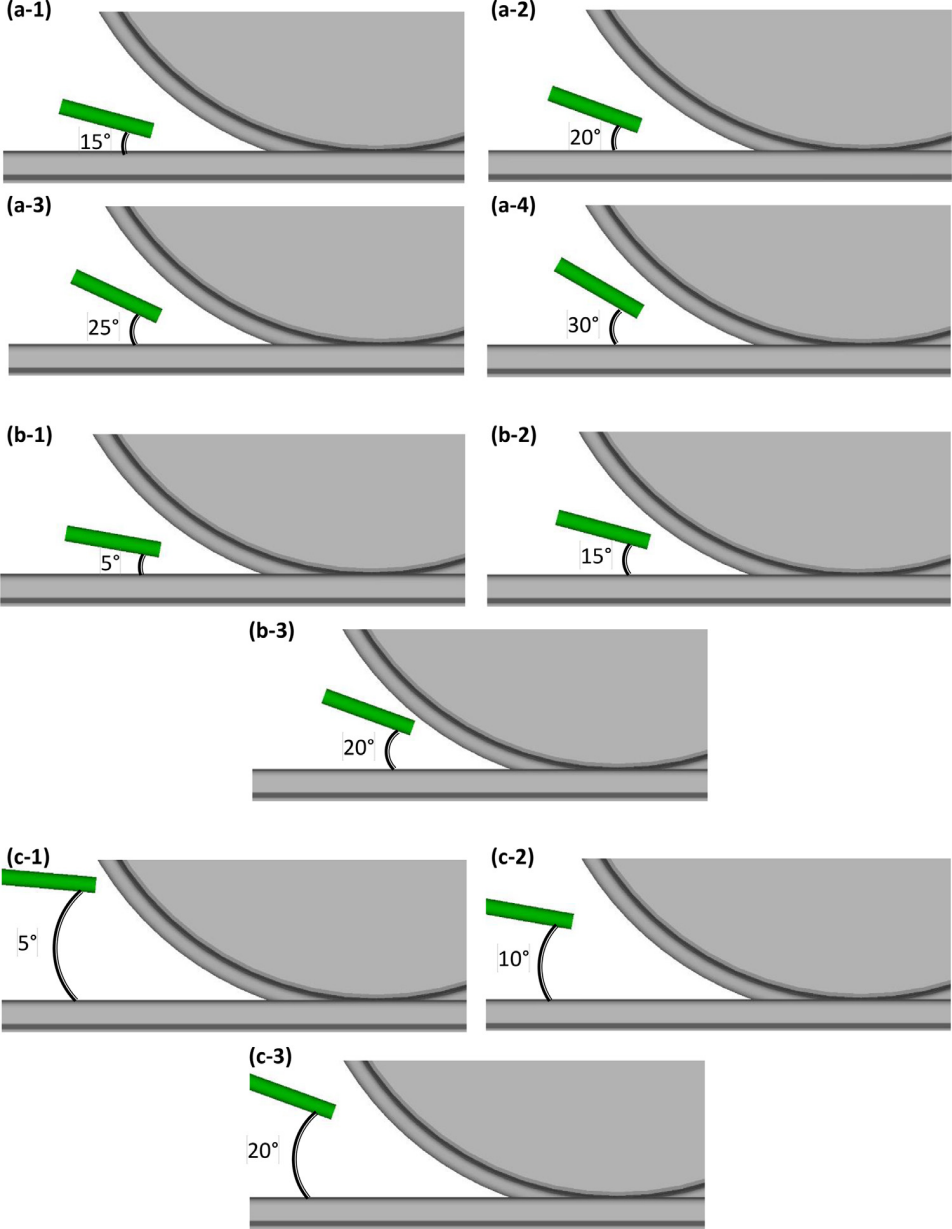
Schematic of sander hose aimed at the **(a)** rail with an angle of **(1)** 15°, **(2)** 20°, **(3)** 25°, and **(4)** 30°, aimed at the **(b)** nip with an angle of **(1)** 10°, **(2)** 15°, and **(3)** 20°, and aimed at the **(c)** wheel with an angle of **(1)** 5°, **(2)** 10°, and **(3)** 20 [1,13].

The sander hose is aimed at the rail with four different angles (relative to the rail surface) of 15°, 20°, 25°, and 30°, aimed at the nip with three different angles of 10°, 15°, and 20°, and aimed at the wheel with three different angles of 5°, 10°, and 20°. Fig. 2 presents schematics of sander hose positioning case studies. Seven granular materials namely the Great Britain (GB) railway grade sand (Garside 10/18), Recycled Crushed Glass (RCG) of three different sizes of 2 mm, 1.18 mm, and 0.6 mm, Coated Alumina, Non-coated Alumina, and Dolomite are used as candidates for alternative rail-sand [11,12]. The translational velocity of the rail is set to ∼0.05 m/s for the case studies of hose’s position and angle and ∼0.1 m/s for the case studies using various granular material. Table 2 presents the test parameters of all the case studies of the geometry of the sanding system including hose’s position and angle relative to the rail surface based on the work of Lewis et al. [1] as well as the granular material used as rail-sand [11].

**Table 2:** Test parameters of all the case studies of the sanding system (geometry and positioning is based on the work of Lewis et al. [1]).

Hose aimed at	Hose aimed at a point on the rail relative to the centre of contact *(mm)*	End of hose distance from contact patch *(mm)*	End of hose height above rail *(mm)*	Hose angle relative to rail *(°)*	Granular material used as rail-sand
Rail	200	350	20	15	GB rail-sand
	200	348	33	20	GB rail-sand
	200	344	45	25	GB rail-sand
	200	340	58	30	GB rail-sand
Nip	100	330	20	10	GB rail-sand
	100	327	40	15	GB rail-sand
	100	323	60	20	GB rail-sand
Wheel	300	440	156	5	GB rail-sand
	300	451	104	10	GB rail-sand
	300	448	131	20	GB rail-sand
Nip	100	330	20	10	GB rail-sand
	100	330	20	10	RCG 2.0 mm
	100	330	20	10	RCG 1.18 mm
	100	330	20	10	RCG 0.6 mm
	100	330	20	10	Dolomite
	100	330	20	10	Uncoated Alumina
	100	330	20	10	Coated Alumina

### 4.3 High speed video recordings

The high-speed videos of the rail-sanding tests are recorded using high-speed cameras. A Phantom V210 camera with the Phantom Camera Control software is utilised for the case studies of hose’s position and aim. Two Daheng Imaging MER-050–560U3M cameras with 5MP 6 MM C-mount lenses and the Galaxy viewer software are used for the case studies of different granular materials to record videos from both front and side views.

For the case studies of the hose position and aim, the high-speed videos are recorded at 750 fps [1] and for the case studies of seven different granular materials used as rail-sand, the high-speed videos are recorded at 560 fps.

For each case study, the experiments were repeated at least three times and videos were recorded for each test. Any data (such as the particles’ entrainment efficiency) can then be averaged through all the tests and the final value reported as the result with determined values for the standard deviation which was below 2.2 % for the particles’ entrainment efficiency.

If during any experiment, the testing platform showed malfunction or unreasonable behaviours or if the value of entrainment efficiency was shown to deviate over ∼3 % compared to the average, the test was removed from the dataset.

For the data extraction form some of the videos in the data set, the first ∼500 frames may need to be omitted as the camera may have been partially blocked by the container collecting the initial sand deposition.

The sander hose positioning case study tests and the various rail-sand case studies were performed by two different people and nearly a decade apart. The results of the tests for the hose aimed at the nip using GB rail-sand confirmed the repeatability of the methodology.

### 4.4 Measuring the particle flow velocity

The velocity of the particle flow can be extracted using any digital Particle Image Velocimetry (PIV) tool such as the MATLAB toolbox called PIVlab [[14], [15], [16]] which was employed here. For more information on the velocity extraction and comparison with the data from numerical simulation refer to [[13], [17]].

**Fig. 3:**
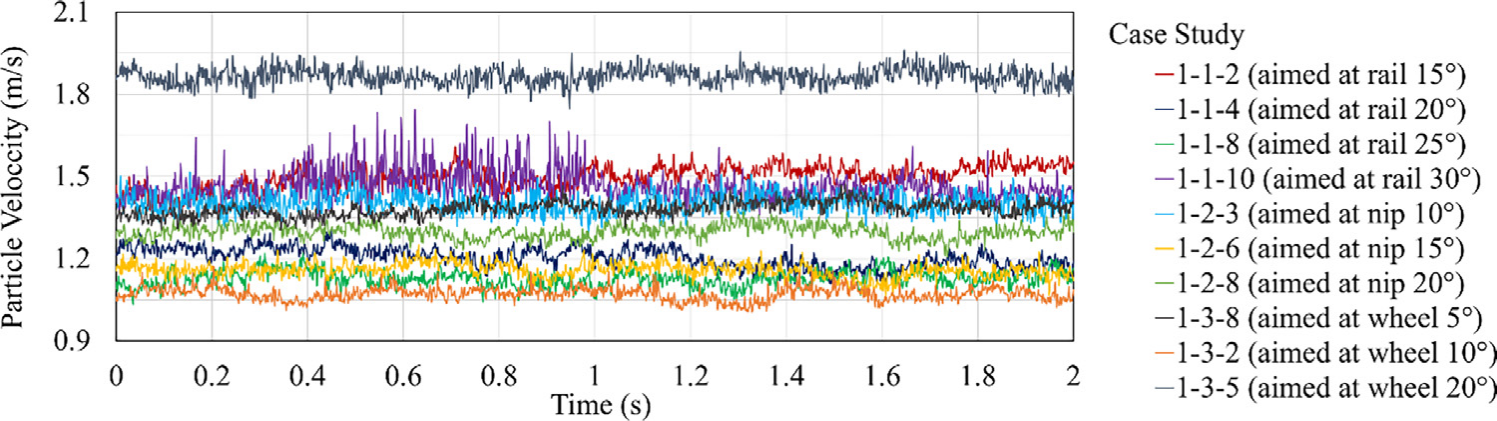
Averaged particle velocity extracted using digital PIV from high-speed videos for case studies of the hose’s position, angle, and aim.

**Fig. 4:**
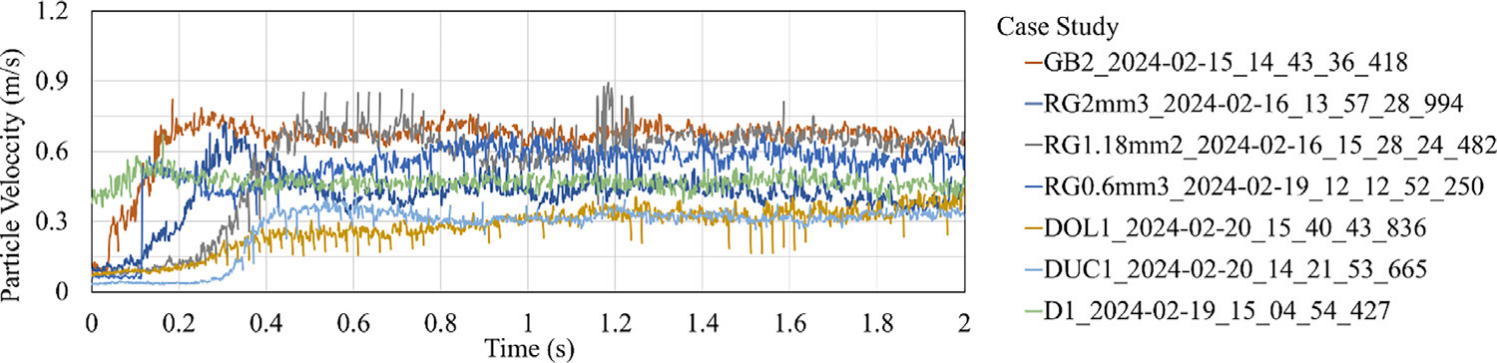
Averaged particle velocity extracted using digital PIV from high-speed videos for case studies of the seven candidate granular materials used as rail-sand.

The extracted PIV data for the case studies of the geometry of the sanding system (hose’s position, angle, and aim) and for the case studies of the granular materials used as rail-sand are presented in Fig. 3, Fig. 4, respectively.

The particle velocities presented in Fig. 3, Fig. 4 are averaged over all the sand particles exiting the sander hose for each time step during the whole duration of the rail-sanding process. The colour of each velocity graph corresponds to a selected case study defined in the legend presented beside each figure.

### 4.5 Measuring the particle flow spread angle

During rail-sanding, the sand particles spread out with an angle as they flow out of the hose. This can be due to a list of factors including the size and shape distribution of sand particles as well as their interactions inside the hopper and while moving through the sander hose. This angle can be measured from the experimental videos using any image/video processing software such as Fiji ImageJ [18] which was used here.

**Fig. 5:**
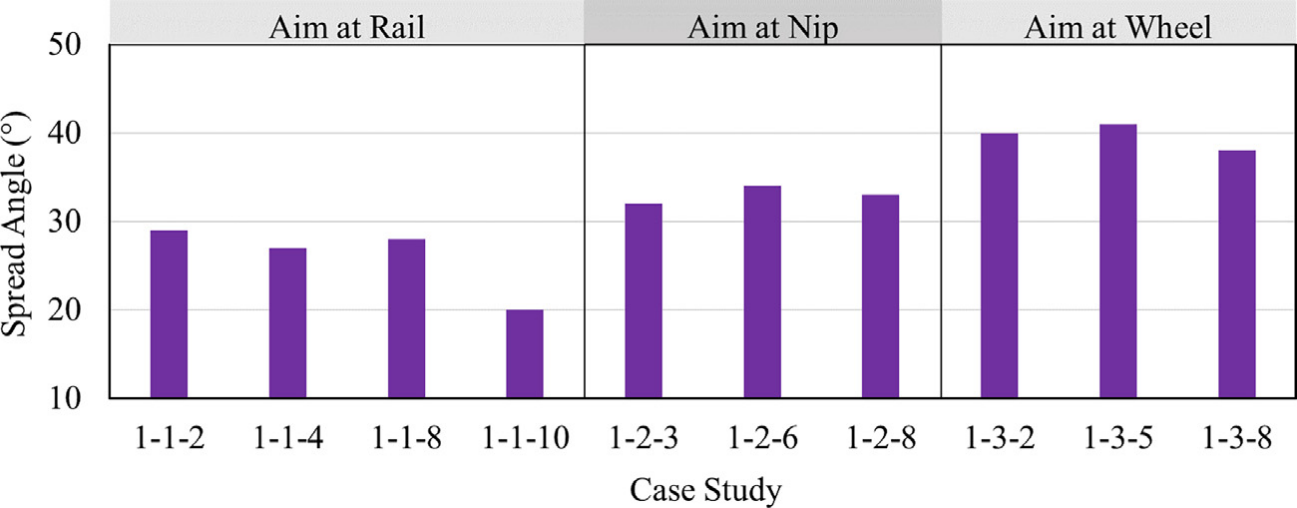
Particle spread angle extracted using image processing from high-speed videos for case studies of the hose’s position, angle, and aim.

The particle spread angles for different case studies of the hose’s position, angle, and aim are presented in Fig. 5. When the sander hose is aimed at the wheel, the vertical distance between the sander hose and the wheel/rail interface is larger compared to when the sander hose is aimed at the nip which results in larger spread angles when the sander hose is aimed at the wheel [1,13].

## Limitations

Full-scale experiments of the rail-sanding can present a more accurate representation of the rail-sanding process compared to other test methods (such as twin-disks test); however, a number of limitations still exist. One limitation is the length of the rail which is much shorter compared to the typical train tracks. Another shortcoming of these tests is the maximum rail speed which is much lower compared to the condition that the actual rail-sanding systems operate at. However, the controlled conditions that the full-scale experiments are performed in can present higher accuracy to study a particular parameter of the sanding systems.

## Ethics Statement

The authors have read and follow the ethical requirements for publication in Data in Brief and confirm that the current work does not involve human subjects, animal experiments, or any data collected from social media platforms.

## CRediT Author Statement

**Sadaf Maramizonouz:** Methodology, Software, Validation, Formal analysis, Investigation, Data Curation, Visualization, Writing - Original Draft, Writing - Review & Editing. **Sadegh Nadimi:** Methodology, Resources, Supervision, Project administration, Funding acquisition, Writing - Review & Editing. **William Skipper:** Methodology, Investigation, Data Curation, Writing - Review & Editing. **Stephen R. Lewis:** Methodology, Investigation, Data Curation, Writing - Review & Editing. **Roger Lewis:** Methodology, Resources, Supervision, Writing - Review & Editing.

## Data Availability

ZenodoHigh-Speed Video Recordings of Wheel-Rail Traction Enhancement Using a Full-Scale Testing Platform (Original data)
ZenodoHigh-Speed Video Recordings of Wheel-Rail Traction Enhancement Using a Full-Scale Testing Platform (Original data)
ZenodoHigh-Speed Video Recordings of Wheel-Rail Traction Enhancement Using a Full-Scale Testing Platform (Original data)

## Footnotes

1 In the railway industry “adhesion” or “adhesion coefficient” is defined as the amount of traction present when the wheel-rail contact enters partial slip. In this paper, the terms are used interchangeably.
